# Supplementary material for: Modeling glioblastoma heterogeneity as a dynamic network of cell states
Source: Mol Syst Biol. 2021 Sep 16;17(9):e10105. doi: 10.15252/msb.202010105 (PMC8444284; doi:10.15252/msb.202010105)
Supplement: Supplementary file 6 — Source Data for Figure 5 [file MSB-17-e10105-s004.zip › Figure5A_sourcedata/GSEA_3071/nosigdb_stateIV.GseaPreranked.1622795164626/DOROSZKO_U3180MG_CLUSTER_3.html]

Details for gene set DOROSZKO\_U3180MG\_CLUSTER\_3[GSEA]

|  || Dataset | state53071 |
| Phenotype | NoPhenotypeAvailable |
| Upregulated in class | na\_neg |
| GeneSet | DOROSZKO\_U3180MG\_CLUSTER\_3 |
| Enrichment Score (ES) | -0.58648753 |
| Normalized Enrichment Score (NES) | -3.763406 |
| Nominal p-value | 0.0 |
| FDR q-value | 0.0 |
| FWER p-Value | 0.0 |
Table: GSEA Results Summary

  

Fig 1: Enrichment plot: DOROSZKO\_U3180MG\_CLUSTER\_3      
 Profile of the Running ES Score & Positions of GeneSet Members on the Rank Ordered List

  

| PROBE | GENE SYMBOL | GENE\_TITLE | RANK IN GENE LIST | RANK METRIC SCORE | RUNNING ES | CORE ENRICHMENT || 1 | INAFM1 |  |  | 107 | 1.515 | -0.0160 | No |
| 2 | MCUB |  |  | 150 | 1.375 | -0.0193 | No |
| 3 | CSRP2 |  |  | 175 | 1.305 | -0.0192 | No |
| 4 | METRN |  |  | 531 | 0.783 | -0.0885 | No |
| 5 | EMP2 |  |  | 732 | 0.642 | -0.1267 | No |
| 6 | GAMT |  |  | 747 | 0.634 | -0.1271 | No |
| 7 | CDCA7 |  |  | 818 | 0.596 | -0.1391 | No |
| 8 | NFIC |  |  | 861 | 0.575 | -0.1455 | No |
| 9 | PGRMC1 |  |  | 1004 | 0.521 | -0.1724 | No |
| 10 | NME4 |  |  | 1055 | 0.504 | -0.1806 | No |
| 11 | OLFM1 |  |  | 1064 | 0.502 | -0.1803 | No |
| 12 | GNG4 |  |  | 1230 | 0.457 | -0.2122 | No |
| 13 | H1FX |  |  | 1238 | 0.454 | -0.2118 | No |
| 14 | ASRGL1 |  |  | 1258 | 0.451 | -0.2140 | No |
| 15 | LRRCC1 |  |  | 1636 | 0.361 | -0.2893 | No |
| 16 | ACTL6A |  |  | 1789 | 0.333 | -0.3190 | No |
| 17 | NABP2 |  |  | 1985 | 0.300 | -0.3575 | No |
| 18 | RBBP4 |  |  | 2000 | 0.297 | -0.3592 | No |
| 19 | SNRNP25 |  |  | 2149 | 0.277 | -0.3883 | No |
| 20 | CARHSP1 |  |  | 2185 | 0.270 | -0.3944 | No |
| 21 | PIN1 |  |  | 2204 | 0.267 | -0.3970 | No |
| 22 | GTF3A |  |  | 2333 | -0.250 | -0.4221 | No |
| 23 | IMMT |  |  | 2380 | -0.256 | -0.4305 | No |
| 24 | MZT2A |  |  | 2381 | -0.256 | -0.4295 | No |
| 25 | SNRPD3 |  |  | 2401 | -0.258 | -0.4324 | No |
| 26 | MRM2 |  |  | 2436 | -0.262 | -0.4383 | No |
| 27 | LMNB1 |  |  | 2487 | -0.268 | -0.4474 | No |
| 28 | VKORC1 |  |  | 2521 | -0.272 | -0.4531 | No |
| 29 | RAD1 |  |  | 2544 | -0.274 | -0.4565 | No |
| 30 | RECQL4 |  |  | 2603 | -0.283 | -0.4673 | No |
| 31 | CMC2 |  |  | 2632 | -0.287 | -0.4719 | No |
| 32 | POP7 |  |  | 2651 | -0.290 | -0.4744 | No |
| 33 | MRPS11 |  |  | 2685 | -0.293 | -0.4800 | No |
| 34 | XRCC6 |  |  | 2696 | -0.294 | -0.4809 | No |
| 35 | AHCY |  |  | 2734 | -0.297 | -0.4873 | No |
| 36 | DEPDC1B |  |  | 2744 | -0.300 | -0.4880 | No |
| 37 | VOPP1 |  |  | 2783 | -0.304 | -0.4946 | No |
| 38 | TMEM237 |  |  | 2811 | -0.307 | -0.4989 | No |
| 39 | PRKDC |  |  | 2815 | -0.307 | -0.4984 | No |
| 40 | RAD54L |  |  | 2855 | -0.313 | -0.5051 | No |
| 41 | BANF1 |  |  | 2865 | -0.315 | -0.5057 | No |
| 42 | PANK2 |  |  | 2869 | -0.315 | -0.5052 | No |
| 43 | ARL6IP6 |  |  | 2877 | -0.316 | -0.5054 | No |
| 44 | RMI2 |  |  | 2893 | -0.318 | -0.5072 | No |
| 45 | FBXO4 |  |  | 2906 | -0.319 | -0.5084 | No |
| 46 | DCK |  |  | 2907 | -0.319 | -0.5072 | No |
| 47 | MCUR1 |  |  | 2924 | -0.321 | -0.5093 | No |
| 48 | HNRNPAB |  |  | 2944 | -0.324 | -0.5119 | No |
| 49 | EZH2 |  |  | 2946 | -0.324 | -0.5109 | No |
| 50 | TIMM10 |  |  | 2963 | -0.328 | -0.5129 | No |
| 51 | SMC1A |  |  | 2968 | -0.328 | -0.5124 | No |
| 52 | UBR7 |  |  | 2985 | -0.331 | -0.5144 | No |
| 53 | WDR54 |  |  | 2987 | -0.331 | -0.5134 | No |
| 54 | GLO1 |  |  | 2996 | -0.332 | -0.5137 | No |
| 55 | FAM161A |  |  | 3009 | -0.333 | -0.5149 | No |
| 56 | HACD3 |  |  | 3013 | -0.334 | -0.5142 | No |
| 57 | SMC4 |  |  | 3035 | -0.337 | -0.5172 | No |
| 58 | MSH6 |  |  | 3051 | -0.339 | -0.5190 | No |
| 59 | MCMBP |  |  | 3056 | -0.339 | -0.5185 | No |
| 60 | UQCC2 |  |  | 3062 | -0.340 | -0.5182 | No |
| 61 | PRTFDC1 |  |  | 3081 | -0.342 | -0.5206 | No |
| 62 | HAUS1 |  |  | 3106 | -0.347 | -0.5242 | No |
| 63 | EIF4A3 |  |  | 3116 | -0.349 | -0.5247 | No |
| 64 | RPA1 |  |  | 3127 | -0.350 | -0.5254 | No |
| 65 | SF3B4 |  |  | 3171 | -0.356 | -0.5328 | No |
| 66 | PSMD14 |  |  | 3196 | -0.359 | -0.5363 | No |
| 67 | ACYP1 |  |  | 3220 | -0.363 | -0.5396 | No |
| 68 | TEDC1 |  |  | 3239 | -0.366 | -0.5418 | No |
| 69 | MCM2 |  |  | 3240 | -0.366 | -0.5404 | No |
| 70 | C5orf34 |  |  | 3251 | -0.368 | -0.5411 | No |
| 71 | PGK1 |  |  | 3257 | -0.369 | -0.5407 | No |
| 72 | P4HB |  |  | 3260 | -0.369 | -0.5397 | No |
| 73 | SAAL1 |  |  | 3290 | -0.374 | -0.5442 | No |
| 74 | POC1A |  |  | 3305 | -0.376 | -0.5456 | No |
| 75 | PKMYT1 |  |  | 3311 | -0.377 | -0.5452 | No |
| 76 | MXD3 |  |  | 3316 | -0.377 | -0.5445 | No |
| 77 | CISD2 |  |  | 3319 | -0.378 | -0.5435 | No |
| 78 | HIRIP3 |  |  | 3328 | -0.379 | -0.5437 | No |
| 79 | MTFR2 |  |  | 3350 | -0.382 | -0.5465 | No |
| 80 | DCTPP1 |  |  | 3414 | -0.393 | -0.5578 | No |
| 81 | RAD51C |  |  | 3415 | -0.393 | -0.5563 | No |
| 82 | HAUS8 |  |  | 3443 | -0.398 | -0.5603 | No |
| 83 | CHAF1B |  |  | 3451 | -0.400 | -0.5602 | No |
| 84 | MNS1 |  |  | 3455 | -0.401 | -0.5593 | No |
| 85 | PSMG2 |  |  | 3459 | -0.401 | -0.5584 | No |
| 86 | AK2 |  |  | 3461 | -0.402 | -0.5571 | No |
| 87 | CCT2 |  |  | 3491 | -0.408 | -0.5614 | No |
| 88 | CENPX |  |  | 3495 | -0.409 | -0.5605 | No |
| 89 | ZNF367 |  |  | 3507 | -0.411 | -0.5611 | No |
| 90 | NDUFA6 |  |  | 3511 | -0.412 | -0.5602 | No |
| 91 | RALY |  |  | 3514 | -0.412 | -0.5590 | No |
| 92 | CDK2 |  |  | 3521 | -0.414 | -0.5587 | No |
| 93 | TFDP1 |  |  | 3526 | -0.415 | -0.5579 | No |
| 94 | NME1 |  |  | 3548 | -0.419 | -0.5606 | No |
| 95 | CHAC2 |  |  | 3558 | -0.420 | -0.5608 | No |
| 96 | MRPL18 |  |  | 3568 | -0.422 | -0.5610 | No |
| 97 | CKAP2 |  |  | 3633 | -0.433 | -0.5724 | No |
| 98 | PPP1CA |  |  | 3654 | -0.436 | -0.5748 | No |
| 99 | SPATA33 |  |  | 3660 | -0.437 | -0.5742 | No |
| 100 | TMEM106C |  |  | 3669 | -0.438 | -0.5741 | No |
| 101 | NUDT5 |  |  | 3687 | -0.442 | -0.5759 | No |
| 102 | CEP57 |  |  | 3694 | -0.443 | -0.5754 | No |
| 103 | ACAT2 |  |  | 3695 | -0.443 | -0.5737 | No |
| 104 | FANCD2 |  |  | 3701 | -0.445 | -0.5731 | No |
| 105 | KDELC2 |  |  | 3706 | -0.447 | -0.5722 | No |
| 106 | RAD18 |  |  | 3717 | -0.448 | -0.5725 | No |
| 107 | CBR3 |  |  | 3745 | -0.454 | -0.5763 | No |
| 108 | HAUS2 |  |  | 3746 | -0.455 | -0.5745 | No |
| 109 | TUBG1 |  |  | 3775 | -0.460 | -0.5785 | No |
| 110 | PRADC1 |  |  | 3777 | -0.461 | -0.5769 | No |
| 111 | GPN3 |  |  | 3790 | -0.464 | -0.5776 | No |
| 112 | DERA |  |  | 3799 | -0.466 | -0.5775 | No |
| 113 | YEATS4 |  |  | 3803 | -0.466 | -0.5763 | No |
| 114 | LMNB2 |  |  | 3817 | -0.469 | -0.5772 | No |
| 115 | KIF22 |  |  | 3832 | -0.472 | -0.5782 | No |
| 116 | GINS4 |  |  | 3843 | -0.474 | -0.5784 | No |
| 117 | IER3IP1 |  |  | 3859 | -0.478 | -0.5797 | No |
| 118 | SUPT16H |  |  | 3872 | -0.481 | -0.5803 | No |
| 119 | TIMELESS |  |  | 3877 | -0.482 | -0.5793 | No |
| 120 | CCND3 |  |  | 3901 | -0.487 | -0.5821 | No |
| 121 | PARP2 |  |  | 3910 | -0.490 | -0.5818 | No |
| 122 | MIS18BP1 |  |  | 3913 | -0.490 | -0.5804 | No |
| 123 | SRPK1 |  |  | 3944 | -0.496 | -0.5846 | Yes |
| 124 | SMC3 |  |  | 3952 | -0.498 | -0.5841 | Yes |
| 125 | TALDO1 |  |  | 3957 | -0.500 | -0.5830 | Yes |
| 126 | DSCC1 |  |  | 3962 | -0.501 | -0.5819 | Yes |
| 127 | CIP2A |  |  | 3968 | -0.502 | -0.5810 | Yes |
| 128 | NUP85 |  |  | 3970 | -0.503 | -0.5793 | Yes |
| 129 | MAGOHB |  |  | 3992 | -0.509 | -0.5817 | Yes |
| 130 | SLBP |  |  | 3994 | -0.509 | -0.5799 | Yes |
| 131 | CENPF |  |  | 4003 | -0.511 | -0.5796 | Yes |
| 132 | ARHGAP11A |  |  | 4007 | -0.513 | -0.5783 | Yes |
| 133 | VPS29 |  |  | 4010 | -0.515 | -0.5767 | Yes |
| 134 | HIST1H1E |  |  | 4011 | -0.515 | -0.5748 | Yes |
| 135 | CCDC15 |  |  | 4019 | -0.518 | -0.5742 | Yes |
| 136 | ATP5MC1 |  |  | 4020 | -0.518 | -0.5722 | Yes |
| 137 | WDR34 |  |  | 4022 | -0.518 | -0.5705 | Yes |
| 138 | PHF19 |  |  | 4041 | -0.523 | -0.5721 | Yes |
| 139 | WDR76 |  |  | 4060 | -0.527 | -0.5738 | Yes |
| 140 | MMS22L |  |  | 4062 | -0.528 | -0.5720 | Yes |
| 141 | ITGB3BP |  |  | 4071 | -0.531 | -0.5716 | Yes |
| 142 | TEX30 |  |  | 4076 | -0.532 | -0.5704 | Yes |
| 143 | ZWILCH |  |  | 4082 | -0.534 | -0.5694 | Yes |
| 144 | TUBB |  |  | 4083 | -0.534 | -0.5673 | Yes |
| 145 | EXO1 |  |  | 4094 | -0.539 | -0.5673 | Yes |
| 146 | E2F8 |  |  | 4104 | -0.542 | -0.5671 | Yes |
| 147 | CACYBP |  |  | 4109 | -0.542 | -0.5658 | Yes |
| 148 | SSRP1 |  |  | 4112 | -0.544 | -0.5642 | Yes |
| 149 | CENPJ |  |  | 4148 | -0.555 | -0.5692 | Yes |
| 150 | EEF1B2 |  |  | 4153 | -0.555 | -0.5679 | Yes |
| 151 | FOXM1 |  |  | 4158 | -0.556 | -0.5666 | Yes |
| 152 | COX5A |  |  | 4172 | -0.560 | -0.5671 | Yes |
| 153 | PPIF |  |  | 4178 | -0.561 | -0.5660 | Yes |
| 154 | CENPQ |  |  | 4190 | -0.564 | -0.5661 | Yes |
| 155 | SPC24 |  |  | 4194 | -0.565 | -0.5645 | Yes |
| 156 | CEP135 |  |  | 4203 | -0.568 | -0.5640 | Yes |
| 157 | MRPL17 |  |  | 4210 | -0.569 | -0.5630 | Yes |
| 158 | FAF1 |  |  | 4211 | -0.570 | -0.5609 | Yes |
| 159 | PSMC3 |  |  | 4224 | -0.573 | -0.5611 | Yes |
| 160 | MIS18A |  |  | 4230 | -0.575 | -0.5600 | Yes |
| 161 | ANP32B |  |  | 4235 | -0.576 | -0.5586 | Yes |
| 162 | COMMD4 |  |  | 4242 | -0.577 | -0.5576 | Yes |
| 163 | HMGB2 |  |  | 4243 | -0.578 | -0.5554 | Yes |
| 164 | BUB3 |  |  | 4249 | -0.580 | -0.5542 | Yes |
| 165 | SNRPD1 |  |  | 4254 | -0.582 | -0.5528 | Yes |
| 166 | EEF1E1 |  |  | 4257 | -0.583 | -0.5510 | Yes |
| 167 | SIVA1 |  |  | 4263 | -0.586 | -0.5498 | Yes |
| 168 | SUMO3 |  |  | 4265 | -0.587 | -0.5478 | Yes |
| 169 | PPIH |  |  | 4268 | -0.587 | -0.5459 | Yes |
| 170 | ARPC5L |  |  | 4269 | -0.588 | -0.5437 | Yes |
| 171 | MCM6 |  |  | 4273 | -0.590 | -0.5421 | Yes |
| 172 | MTHFD1 |  |  | 4274 | -0.590 | -0.5398 | Yes |
| 173 | DEGS1 |  |  | 4281 | -0.592 | -0.5388 | Yes |
| 174 | EXOSC8 |  |  | 4293 | -0.596 | -0.5387 | Yes |
| 175 | SLF1 |  |  | 4294 | -0.596 | -0.5365 | Yes |
| 176 | FARSB |  |  | 4299 | -0.597 | -0.5350 | Yes |
| 177 | BRIP1 |  |  | 4301 | -0.598 | -0.5329 | Yes |
| 178 | SCCPDH |  |  | 4318 | -0.602 | -0.5339 | Yes |
| 179 | KIF15 |  |  | 4324 | -0.603 | -0.5326 | Yes |
| 180 | ASF1B |  |  | 4328 | -0.606 | -0.5309 | Yes |
| 181 | POLE3 |  |  | 4336 | -0.610 | -0.5300 | Yes |
| 182 | SGO1 |  |  | 4339 | -0.611 | -0.5281 | Yes |
| 183 | RFC4 |  |  | 4340 | -0.611 | -0.5258 | Yes |
| 184 | RCC1 |  |  | 4341 | -0.612 | -0.5235 | Yes |
| 185 | POLE4 |  |  | 4360 | -0.621 | -0.5248 | Yes |
| 186 | KPNA2 |  |  | 4361 | -0.621 | -0.5224 | Yes |
| 187 | MRPL37 |  |  | 4366 | -0.623 | -0.5208 | Yes |
| 188 | KNL1 |  |  | 4367 | -0.624 | -0.5185 | Yes |
| 189 | LIG1 |  |  | 4368 | -0.624 | -0.5161 | Yes |
| 190 | RAB23 |  |  | 4372 | -0.625 | -0.5143 | Yes |
| 191 | RBBP8 |  |  | 4375 | -0.627 | -0.5123 | Yes |
| 192 | POLA2 |  |  | 4379 | -0.627 | -0.5106 | Yes |
| 193 | DNMT1 |  |  | 4388 | -0.632 | -0.5098 | Yes |
| 194 | TPRKB |  |  | 4399 | -0.635 | -0.5094 | Yes |
| 195 | FANCA |  |  | 4403 | -0.635 | -0.5076 | Yes |
| 196 | ALYREF |  |  | 4406 | -0.638 | -0.5056 | Yes |
| 197 | CSE1L |  |  | 4419 | -0.642 | -0.5056 | Yes |
| 198 | C19orf48 |  |  | 4427 | -0.644 | -0.5045 | Yes |
| 199 | DONSON |  |  | 4444 | -0.652 | -0.5053 | Yes |
| 200 | ITGB1BP1 |  |  | 4445 | -0.653 | -0.5028 | Yes |
| 201 | SAC3D1 |  |  | 4462 | -0.660 | -0.5036 | Yes |
| 202 | DDX39A |  |  | 4467 | -0.661 | -0.5019 | Yes |
| 203 | RBL1 |  |  | 4469 | -0.662 | -0.4995 | Yes |
| 204 | SHCBP1 |  |  | 4473 | -0.667 | -0.4976 | Yes |
| 205 | EIF4EBP1 |  |  | 4474 | -0.667 | -0.4951 | Yes |
| 206 | RPA3 |  |  | 4482 | -0.670 | -0.4939 | Yes |
| 207 | BUB1B |  |  | 4483 | -0.670 | -0.4914 | Yes |
| 208 | PDCD5 |  |  | 4485 | -0.672 | -0.4890 | Yes |
| 209 | MASTL |  |  | 4490 | -0.673 | -0.4873 | Yes |
| 210 | PSMB9 |  |  | 4494 | -0.677 | -0.4853 | Yes |
| 211 | H2AFX |  |  | 4495 | -0.677 | -0.4828 | Yes |
| 212 | PA2G4 |  |  | 4497 | -0.678 | -0.4804 | Yes |
| 213 | NUDT15 |  |  | 4506 | -0.684 | -0.4794 | Yes |
| 214 | SNRPF |  |  | 4514 | -0.687 | -0.4782 | Yes |
| 215 | DHFR |  |  | 4517 | -0.688 | -0.4760 | Yes |
| 216 | NCAPH2 |  |  | 4534 | -0.698 | -0.4766 | Yes |
| 217 | MND1 |  |  | 4538 | -0.699 | -0.4745 | Yes |
| 218 | RFC5 |  |  | 4543 | -0.701 | -0.4727 | Yes |
| 219 | CCNE2 |  |  | 4564 | -0.714 | -0.4740 | Yes |
| 220 | DTYMK |  |  | 4566 | -0.715 | -0.4715 | Yes |
| 221 | NCAPG |  |  | 4568 | -0.716 | -0.4690 | Yes |
| 222 | DBF4 |  |  | 4570 | -0.717 | -0.4665 | Yes |
| 223 | CTPS1 |  |  | 4572 | -0.718 | -0.4639 | Yes |
| 224 | MGME1 |  |  | 4573 | -0.722 | -0.4612 | Yes |
| 225 | TUBB4B |  |  | 4575 | -0.722 | -0.4587 | Yes |
| 226 | IKBIP |  |  | 4584 | -0.727 | -0.4575 | Yes |
| 227 | PRIM2 |  |  | 4585 | -0.728 | -0.4547 | Yes |
| 228 | RFC2 |  |  | 4588 | -0.729 | -0.4524 | Yes |
| 229 | PBK |  |  | 4593 | -0.730 | -0.4504 | Yes |
| 230 | CEP152 |  |  | 4599 | -0.733 | -0.4486 | Yes |
| 231 | CKS2 |  |  | 4600 | -0.733 | -0.4458 | Yes |
| 232 | TMEM14C |  |  | 4608 | -0.737 | -0.4445 | Yes |
| 233 | SVIP |  |  | 4609 | -0.737 | -0.4417 | Yes |
| 234 | CCT4 |  |  | 4613 | -0.740 | -0.4395 | Yes |
| 235 | BIRC5 |  |  | 4618 | -0.745 | -0.4374 | Yes |
| 236 | CKLF |  |  | 4619 | -0.746 | -0.4346 | Yes |
| 237 | AP2S1 |  |  | 4621 | -0.747 | -0.4320 | Yes |
| 238 | RACGAP1 |  |  | 4623 | -0.748 | -0.4293 | Yes |
| 239 | PARPBP |  |  | 4625 | -0.749 | -0.4267 | Yes |
| 240 | E2F1 |  |  | 4637 | -0.760 | -0.4260 | Yes |
| 241 | RPA2 |  |  | 4638 | -0.760 | -0.4231 | Yes |
| 242 | NCAPD3 |  |  | 4640 | -0.760 | -0.4204 | Yes |
| 243 | DUT |  |  | 4642 | -0.762 | -0.4177 | Yes |
| 244 | USP1 |  |  | 4649 | -0.763 | -0.4160 | Yes |
| 245 | SGO2 |  |  | 4650 | -0.764 | -0.4131 | Yes |
| 246 | NUSAP1 |  |  | 4654 | -0.765 | -0.4108 | Yes |
| 247 | FANCI |  |  | 4665 | -0.772 | -0.4099 | Yes |
| 248 | GGCT |  |  | 4666 | -0.772 | -0.4070 | Yes |
| 249 | PSMA3 |  |  | 4667 | -0.773 | -0.4040 | Yes |
| 250 | SLC43A3 |  |  | 4668 | -0.775 | -0.4011 | Yes |
| 251 | NUDC |  |  | 4670 | -0.776 | -0.3983 | Yes |
| 252 | MSH2 |  |  | 4673 | -0.781 | -0.3958 | Yes |
| 253 | CENPH |  |  | 4679 | -0.786 | -0.3938 | Yes |
| 254 | KPNB1 |  |  | 4681 | -0.786 | -0.3910 | Yes |
| 255 | COPS3 |  |  | 4694 | -0.791 | -0.3905 | Yes |
| 256 | MYBL1 |  |  | 4697 | -0.795 | -0.3878 | Yes |
| 257 | CDC45 |  |  | 4702 | -0.798 | -0.3856 | Yes |
| 258 | MCM7 |  |  | 4704 | -0.800 | -0.3828 | Yes |
| 259 | SAE1 |  |  | 4711 | -0.801 | -0.3809 | Yes |
| 260 | WDHD1 |  |  | 4713 | -0.803 | -0.3781 | Yes |
| 261 | TCF19 |  |  | 4719 | -0.807 | -0.3760 | Yes |
| 262 | TRIP13 |  |  | 4721 | -0.808 | -0.3732 | Yes |
| 263 | GTSE1 |  |  | 4722 | -0.808 | -0.3701 | Yes |
| 264 | KIF4A |  |  | 4727 | -0.812 | -0.3678 | Yes |
| 265 | CDCA2 |  |  | 4732 | -0.816 | -0.3655 | Yes |
| 266 | SHMT2 |  |  | 4737 | -0.820 | -0.3632 | Yes |
| 267 | CCT5 |  |  | 4740 | -0.822 | -0.3605 | Yes |
| 268 | RANBP1 |  |  | 4741 | -0.823 | -0.3573 | Yes |
| 269 | CHAF1A |  |  | 4751 | -0.828 | -0.3560 | Yes |
| 270 | POLD3 |  |  | 4752 | -0.830 | -0.3529 | Yes |
| 271 | MELK |  |  | 4758 | -0.835 | -0.3507 | Yes |
| 272 | CHEK1 |  |  | 4764 | -0.837 | -0.3485 | Yes |
| 273 | C21orf58 |  |  | 4767 | -0.838 | -0.3458 | Yes |
| 274 | CKAP2L |  |  | 4769 | -0.839 | -0.3428 | Yes |
| 275 | ARPC1B |  |  | 4771 | -0.840 | -0.3398 | Yes |
| 276 | VRK1 |  |  | 4773 | -0.842 | -0.3368 | Yes |
| 277 | NUF2 |  |  | 4782 | -0.847 | -0.3352 | Yes |
| 278 | ASPM |  |  | 4789 | -0.851 | -0.3332 | Yes |
| 279 | TMPO |  |  | 4790 | -0.851 | -0.3299 | Yes |
| 280 | MCM5 |  |  | 4792 | -0.853 | -0.3269 | Yes |
| 281 | CENPN |  |  | 4796 | -0.858 | -0.3242 | Yes |
| 282 | GINS1 |  |  | 4804 | -0.871 | -0.3223 | Yes |
| 283 | LRR1 |  |  | 4805 | -0.873 | -0.3190 | Yes |
| 284 | SKA1 |  |  | 4808 | -0.873 | -0.3161 | Yes |
| 285 | NCAPG2 |  |  | 4809 | -0.874 | -0.3128 | Yes |
| 286 | CDK4 |  |  | 4810 | -0.874 | -0.3094 | Yes |
| 287 | CDT1 |  |  | 4813 | -0.880 | -0.3065 | Yes |
| 288 | PRIM1 |  |  | 4815 | -0.881 | -0.3033 | Yes |
| 289 | HELLS |  |  | 4819 | -0.882 | -0.3006 | Yes |
| 290 | HSPB11 |  |  | 4820 | -0.882 | -0.2972 | Yes |
| 291 | MCM8 |  |  | 4821 | -0.888 | -0.2939 | Yes |
| 292 | XRCC2 |  |  | 4823 | -0.890 | -0.2907 | Yes |
| 293 | KIF23 |  |  | 4825 | -0.891 | -0.2875 | Yes |
| 294 | MCM3 |  |  | 4834 | -0.901 | -0.2857 | Yes |
| 295 | MKI67 |  |  | 4836 | -0.902 | -0.2825 | Yes |
| 296 | NCAPH |  |  | 4842 | -0.908 | -0.2800 | Yes |
| 297 | KIFC1 |  |  | 4844 | -0.911 | -0.2767 | Yes |
| 298 | PRC1 |  |  | 4846 | -0.912 | -0.2735 | Yes |
| 299 | SMC2 |  |  | 4847 | -0.912 | -0.2700 | Yes |
| 300 | UHRF1 |  |  | 4859 | -0.924 | -0.2687 | Yes |
| 301 | DSN1 |  |  | 4864 | -0.929 | -0.2660 | Yes |
| 302 | ORC6 |  |  | 4865 | -0.931 | -0.2625 | Yes |
| 303 | JPT2 |  |  | 4871 | -0.938 | -0.2599 | Yes |
| 304 | GMNN |  |  | 4873 | -0.939 | -0.2565 | Yes |
| 305 | CDCA3 |  |  | 4874 | -0.940 | -0.2530 | Yes |
| 306 | CENPM |  |  | 4881 | -0.947 | -0.2506 | Yes |
| 307 | RFC3 |  |  | 4887 | -0.954 | -0.2480 | Yes |
| 308 | GINS2 |  |  | 4897 | -0.962 | -0.2461 | Yes |
| 309 | TACC3 |  |  | 4900 | -0.966 | -0.2429 | Yes |
| 310 | CDCA4 |  |  | 4902 | -0.967 | -0.2394 | Yes |
| 311 | RTKN2 |  |  | 4904 | -0.970 | -0.2359 | Yes |
| 312 | KDELR2 |  |  | 4909 | -0.976 | -0.2330 | Yes |
| 313 | FBXO5 |  |  | 4910 | -0.978 | -0.2293 | Yes |
| 314 | SKA3 |  |  | 4914 | -0.981 | -0.2261 | Yes |
| 315 | FAM111A |  |  | 4915 | -0.981 | -0.2224 | Yes |
| 316 | DNAJC9 |  |  | 4916 | -0.982 | -0.2187 | Yes |
| 317 | H2AFZ |  |  | 4919 | -0.983 | -0.2153 | Yes |
| 318 | SNRPB |  |  | 4931 | -0.996 | -0.2138 | Yes |
| 319 | PSMC3IP |  |  | 4933 | -0.997 | -0.2102 | Yes |
| 320 | ATAD5 |  |  | 4934 | -0.998 | -0.2064 | Yes |
| 321 | BRCA1 |  |  | 4938 | -1.002 | -0.2032 | Yes |
| 322 | TUBB6 |  |  | 4939 | -1.003 | -0.1994 | Yes |
| 323 | CTNNAL1 |  |  | 4945 | -1.007 | -0.1966 | Yes |
| 324 | MCM4 |  |  | 4947 | -1.011 | -0.1929 | Yes |
| 325 | PSMG1 |  |  | 4955 | -1.025 | -0.1904 | Yes |
| 326 | CLIC1 |  |  | 4964 | -1.035 | -0.1881 | Yes |
| 327 | DTL |  |  | 4970 | -1.042 | -0.1852 | Yes |
| 328 | CENPW |  |  | 4978 | -1.056 | -0.1826 | Yes |
| 329 | ATAD2 |  |  | 4980 | -1.058 | -0.1788 | Yes |
| 330 | RRM1 |  |  | 4990 | -1.074 | -0.1765 | Yes |
| 331 | UBE2T |  |  | 5000 | -1.091 | -0.1742 | Yes |
| 332 | MAD2L1 |  |  | 5007 | -1.105 | -0.1712 | Yes |
| 333 | SPC25 |  |  | 5012 | -1.112 | -0.1678 | Yes |
| 334 | FEN1 |  |  | 5014 | -1.113 | -0.1637 | Yes |
| 335 | AURKB |  |  | 5016 | -1.118 | -0.1597 | Yes |
| 336 | TK1 |  |  | 5020 | -1.126 | -0.1560 | Yes |
| 337 | IDH2 |  |  | 5022 | -1.133 | -0.1519 | Yes |
| 338 | CDCA5 |  |  | 5023 | -1.133 | -0.1476 | Yes |
| 339 | TUBA1C |  |  | 5027 | -1.149 | -0.1438 | Yes |
| 340 | KIF2C |  |  | 5028 | -1.150 | -0.1394 | Yes |
| 341 | TOP2A |  |  | 5029 | -1.152 | -0.1351 | Yes |
| 342 | MYBL2 |  |  | 5041 | -1.172 | -0.1328 | Yes |
| 343 | PFKP |  |  | 5042 | -1.173 | -0.1284 | Yes |
| 344 | TPX2 |  |  | 5049 | -1.184 | -0.1251 | Yes |
| 345 | CCNA2 |  |  | 5051 | -1.186 | -0.1208 | Yes |
| 346 | DEPDC1 |  |  | 5053 | -1.193 | -0.1164 | Yes |
| 347 | FAM111B |  |  | 5054 | -1.199 | -0.1119 | Yes |
| 348 | NDC80 |  |  | 5055 | -1.200 | -0.1073 | Yes |
| 349 | PCNA |  |  | 5060 | -1.214 | -0.1035 | Yes |
| 350 | MCM10 |  |  | 5066 | -1.222 | -0.0999 | Yes |
| 351 | ESCO2 |  |  | 5067 | -1.222 | -0.0952 | Yes |
| 352 | BRCA2 |  |  | 5069 | -1.222 | -0.0908 | Yes |
| 353 | FUCA2 |  |  | 5070 | -1.227 | -0.0861 | Yes |
| 354 | CENPK |  |  | 5091 | -1.267 | -0.0853 | Yes |
| 355 | SLC20A1 |  |  | 5094 | -1.277 | -0.0809 | Yes |
| 356 | UBE2C |  |  | 5106 | -1.314 | -0.0781 | Yes |
| 357 | CDC6 |  |  | 5107 | -1.314 | -0.0731 | Yes |
| 358 | SMS |  |  | 5109 | -1.315 | -0.0683 | Yes |
| 359 | CCDC34 |  |  | 5111 | -1.316 | -0.0635 | Yes |
| 360 | ZWINT |  |  | 5115 | -1.321 | -0.0591 | Yes |
| 361 | CKS1B |  |  | 5121 | -1.338 | -0.0550 | Yes |
| 362 | HJURP |  |  | 5125 | -1.344 | -0.0505 | Yes |
| 363 | RAD51AP1 |  |  | 5133 | -1.375 | -0.0467 | Yes |
| 364 | TYMS |  |  | 5156 | -1.477 | -0.0456 | Yes |
| 365 | CENPU |  |  | 5159 | -1.486 | -0.0403 | Yes |
| 366 | DIAPH3 |  |  | 5161 | -1.488 | -0.0349 | Yes |
| 367 | CDK1 |  |  | 5164 | -1.512 | -0.0295 | Yes |
| 368 | PHGDH |  |  | 5170 | -1.528 | -0.0247 | Yes |
| 369 | PLP2 |  |  | 5176 | -1.552 | -0.0198 | Yes |
| 370 | CLSPN |  |  | 5202 | -1.663 | -0.0186 | Yes |
| 371 | PCLAF |  |  | 5209 | -1.698 | -0.0133 | Yes |
| 372 | HMGA1 |  |  | 5220 | -1.792 | -0.0086 | Yes |
| 373 | HIST1H1D |  |  | 5231 | -1.871 | -0.0035 | Yes |
| 374 | RRM2 |  |  | 5232 | -1.875 | 0.0037 | Yes |
| 375 | HIST1H4C |  |  | 5249 | -2.081 | 0.0083 | Yes |
Table: GSEA details [plain text format]

  

Fig 2: DOROSZKO\_U3180MG\_CLUSTER\_3: Random ES distribution      
 Gene set null distribution of ES for **DOROSZKO\_U3180MG\_CLUSTER\_3**
